# Power of maximum HLOD tests to detect linkage to obesity genes

**DOI:** 10.1186/1471-2156-4-S1-S16

**Published:** 2003-12-31

**Authors:** Yun Joo Yoo, Yanling Huo, Yuming Ning, Derek Gordon, Stephen Finch, Nancy R Mendell

**Affiliations:** 1 Department of Applied Mathematics and Statistics, State University of New York at Stony Brook, Stony Brook, New York, USA; 2 Chung Yu College of Business Administration, Keelung, Taiwan; 3 Laboratory of Statistical Genetics, The Rockefeller University, New York, New York, USA

## Abstract

**Background:**

We investigate the power of heterogeneity LOD test to detect linkage when a trait is determined by several major genes using Genetic Analysis Workshop 13 simulated data. We consider three traits, two of which are disease-causing traits: 1) the rate of change in body mass index (BMI); and 2) the maximum BMI; and 3) the disease itself (hypertension). Of interest is the power of "HLOD2", the maximum heterogeneity LOD obtained upon maximizing over the two genetic models.

**Results:**

Using a trait phenotype Obesity Slope, we observe that the power to detect the two markers closest to the two genes (S1, S2) at the 0.05 level using HLOD2 is 13% and 10%. The power of HLOD2 for Max BMI phenotype is 12% and 9%. The corresponding values for the Hypertension phenotype are 8% and 6%.

**Conclusion:**

The power to detect linkage to the slope genes is quite low. But the power using disease-related traits as a phenotype is greater than the power using the disease (hypertension) phenotype.

## Background

One of the issues in analysis of complex diseases is the power of considering an disease related trait as a phenotype than the disease itself [1]. Another issue considered here is the method of analysis, which allows for heterogeneity when one or more major genes determine a trait [2]. The Genetic Analysis Workshop 13 (GAW13) simulated longitudinal data on obesity provide an example of a disease-related phenotype. According to the simulation model, the Obesity Slope, the rate of increase in BMI (body mass index) over time is determined by only two major genes (S1, S2). Also, Obesity Slope in turn is a risk factor for hypertension, and it is also implied in the simulation model that these two genes for obesity are in fact two of many hypertension-causing genes.

In this work, we want to investigate the power of HLOD maximized over two genetic models (dominant and recessive) to detect linkage with the Obesity Slope phenotype and also with the hypertension phenotype, i.e., the disease itself. We also consider another trait, the individual's maximum observed BMI (Max BMI), which is related to both obesity and hypertension.

## Methods

### Definition of disease phenotypes

The first phenotype considered is Obesity Slope. For each individual, we regressed BMI values on log of age values at each time where the BMI was recorded, using the following model: E(BMI _ij_) = α_j _+ β_j_log_10_(X_ij_), for i = 1,2,..., t_j_.

Here X_ij _denotes the j^th ^individual's age at the i^th ^measurement; BMI_ij _denotes the j^th ^individual's BMI at age X_ij_; and t_j _denotes the number of time points for which we have BMI values on the j^th ^individual. The value of BMI_ij _is computed, again following the model described in the simulation as BMI_ij _= W_ij_/H_ij_^2^, where W_ij _denotes j^th ^individual's weight in kilograms and H_ij _denotes the j^th ^individual's height in meters at the i^th ^time point observed. Ordinary least-squares regression resulted in estimates of β_j _for each of our subjects. We treated an individual as "missing" slope information if he had too few measurements. Individuals in Cohort 1 with less than three measurements were treated as missing. Individuals in Cohort 2 with less than two measurements were treated as missing. Cohort 1 has 21 time points with two-year intervals and Cohort 2 has five time points with eight- or four-year intervals. We then defined individuals as "Obese" if β_j _≥ b_90 _and "Normal" if β_j _< b_90_, where b_90 _denotes the 90^th ^percentile of the slopes observed in each replicate being analyzed.

The second disease-related phenotype considered here is the maximum of j^th ^individual's BMI over all ages (Max BMI). We defined individuals as "High" if Max BMI ≥ m_90 _and "Normal" if Max BMI < m_90_, where m_90 _denotes the 90th percentile of the Max BMI values observed in each replicate being analyzed. The third phenotype is "Hypertension." Individuals defined as "affected" are those with diastolic BP ≥ 90 and/or systolic BP ≥ 140, and "Normal" are those having diastolic BP < 90 and systolic BP < 140.

### Methods of linkage analysis

Single-point linkage analysis was done for the two markers closest to the two genes S1 and S2, and for five unlinked markers. One marker linked to S1 is 11g6 and the other, linked to S2, is 7g7. We also considered five unlinked markers on chromosomes 2, 4, 6, 8, and 10 (2g5, 4g4, 6g3, 8g2, 10g1), on which none of the disease genes were located.

We used the linkage test specified on Abreu et al. [3]. We obtained a value "HLOD-D" by maximizing over all values of the recombination fraction and heterogeneity parameter assuming disease allele frequency 0.01 and dominance with penetrance 0.5. Then we obtained a value "HLOD-R" by maximizing over all values of the recombination fraction and heterogeneity parameter assuming disease allele frequency 0.01 and recessive model with penetrance 0.5. The test statistic HLOD2 was obtained as the maximum of HLOD-D and HLOD-R. Also, for the comparison, we considered LOD2, the maximum of LOD-D and LOD-R using the same two analysis models without heterogeneity parameter. To calculate HLOD values, we used HOMOG program combining with LINKAGE package.

## Results and Discussion

With the results of analysis of five unlinked markers using the Obesity Slope phenotype, the empirical critical values for a 0.05 level test are 0.947 for HLOD2 and 0.730 for LOD2 from the null distribution of size *N *= 500. Then we obtained power values for the three phenotypes by computing the percentage of replicates with higher HLOD2 (or LOD2) values than the critical value in all 100 replicates. Highest power is 13% for Obesity Slope phenotype at the 11g6 marker. Power values for three phenotypes are presented in the Table 1.

**Figure 1:**
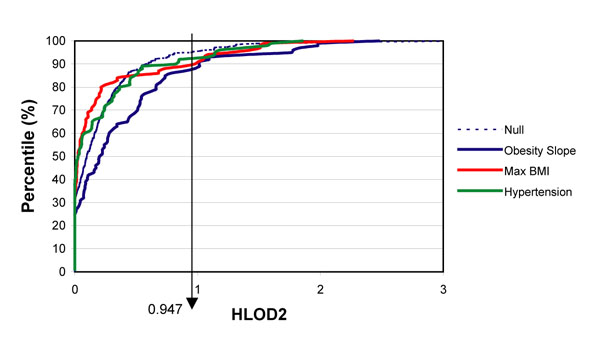
**Distribution of HLOD2 scores of three phenotypes with marker 11g6. **With the critical value 0.947 obtained from the 95% percentile of null distribution (dotted line), Obesity Slope (blue line) has power of 13%, Max BMI (red line) has power of 12%, and Hypertension (green line) has power of 8%.

The Obesity Slope phenotype resulted in the highest power. The second highest is the Max BMI (Figure 1). At a certain level it is not surprising that the Max BMI gives closer results to the Obesity Slope phenotype than Hypertension does. There is a strong association between being in the top 10% for the Obesity Slope and Max BMI phenotypes (χ^2 ^= 19.5). On the other hand, there is no association in this sample between being in the top 10% for the Slope Phenotype and having hypertension (χ^2 ^= 2.66).

There are several possible explanations for the low power to detect linkage observed here. The most likely one is that we lose power by dichotomizing the values rather than analyzing these traits as quantitative phenotypes using model-free methods. Second, even though we are focusing on the slope itself, which is the direct product of the gene, we do have error in our estimate of an individuals' slope due to the combination of random noises in the simulations models.

## Conclusions

The overall power values for HLOD2 analysis are quiet low in the case of the analysis of dichotomized BMI slope values and dichotomized BMI values. However, they are higher than those obtained using the hypertension phenotype.

## Figures and Tables

**Table 1:** Power of HLOD2 and LOD2 test for three phenotypes Critical values of 0.947 for HLOD2 and 0.730 for LOD2 are used for both of markers.

Marker	11g6 (S1)		7g7 (S2)	
		
Phenotype	HLOD2	LOD2	HLOD2	LOD2
Obesity Slope	13%	10%	10%	10%
Max BMI	12%	11%	9%	9%
Hypertension	8%	8%	6%	4%
